# Clinical Incidence and Characteristics of Newly Diagnosed Type 1 Diabetes in Chinese Children and Adolescents: A Nationwide Registry Study of 34 Medical Centers

**DOI:** 10.3389/fped.2022.888370

**Published:** 2022-06-15

**Authors:** Guo-Hua Li, Ke Huang, Guan-Ping Dong, Jian-Wei Zhang, Chun-Xiu Gong, Fei-Hong Luo, Xiao-Ping Luo, Chun-Lin Wang, Min Zhu, Pin Li, Ling Wang, Jun-Fen Fu, The T1DM China Study Group for Children Adolescents

**Affiliations:** ^1^The Children's Hospital of the Zhejiang University School of Medicine, National Clinical Research Center for Child Health, Hangzhou, China; ^2^Department of Pediatrics, Shaoxing Maternal and Child Health Care Hospital, Hangzhou, China; ^3^Endocrinology, Genetics, and Metabolism, Beijing Diabetes Center for Children and Adolescents, Medical Genetics Department, Beijing Children's Hospital, Beijing, China; ^4^Department of Pediatric Endocrinology and Inherited Metabolic Diseases, Children's Hospital of Fudan University, Shanghai, China; ^5^Department of Pediatrics, Tongji Hospital, Tongji Medical College, Huazhong University of Science and Technology, Wuhan, China; ^6^Department of Pediatric, The First Affiliated Hospital of Zhejiang University School of Medicine, Hangzhou, China; ^7^Department of Endocrinology, Children's Hospital of Chongqing Medical University, Chongqing, China; ^8^Department of Endocrinology, Shanghai Children's Hospital of Shanghai Jiao Tong University, Shanghai, China; ^9^Laboratory for Translational Genetics, Department of Human Genetics, KU Leuven, Leuven, Belgium

**Keywords:** type 1 diabetes, incidence, diabetic ketoacidosis, β-cell function, children and adolescences

## Abstract

**Objective:**

To investigate the clinical incidence and characteristics of type 1 diabetes mellitus (T1DM) of children and adolescents at the time of initial diagnosis in China.

**Methods:**

Data on all pediatric patients with newly diagnosed T1DM were retrospectively collected from 34 medical centers in 25 major cities in China from January 2015 to January 2020. Patients were classified into three age groups: <5 years, 5 to <10 years, and ≥10 years of age. The same patient population was also categorized into diabetic ketoacidosis (DKA) and non-DKA groups based on clinical criteria.

**Results:**

The mean annual clinical incidence of T1DM was 3.16/100,000 from the years 2015 to 2019. A total of 6,544 patients with newly diagnosed T1DM aged 0–16 years (median 7.84 ± 3.8) were studied [ages <5 years (29.3%), 5 to <10 years (38.7%), and ≥10 years (32%)], 52.4% of them were women. In total, 90.5% of the cases were occurred in individuals without a family history. Patients had lower C-peptide (CP) and body mass index (BMI) z scores when compared with healthy children, 41.8% of them had measurable T1DM-related antibodies and 52.7% had DKA. Among all three age groups, the <5 years group had the lowest BMI z score, CP, and glycated hemoglobin (HbA1c) on average, while it had the highest incidence rate of DKA (56.9%). Compared to the non-DKA group, the DKA group was significantly younger, with a lower BMI z score and CP, higher antibody positive rate, HbA1c, and the rate of insulin pump therapy.

**Conclusion:**

The clinical incidence of T1DM in children and adolescents in China was 3.16/100,000. Patients with DKA at the first diagnosis of T1DM have a worse β-cell function. Public health measures for the prevention and treatment of T1DM should focus on preschoolers (aged <5 years) in particular, considering the severity and the highest frequency of DKA in this age group. More efforts should be dedicated to early screening and diagnosis of the T1DM.

## Introduction

Type 1 diabetes mellitus (T1DM) is a chronic autoimmune disease characterized by insulin deficiency and resultant hyperglycemia, accounting for about 90% of diabetes in children and adolescents. It is a major pediatric endocrine disease that endangers children's health. According to the data released by International Diabetes Federation (IDF) in 2019, 1.1 million children and adolescents (<20 years of age) have T1DM around the world, and the number of patients continues to increase (1, 2). The global average increase in incidence has been 3–4% per year over the past decades, with an especially steeper rise in low-incidence countries (3). The incidence of T1DM remains unstable in China, a longitudinal population-based registry study showed that it significantly increased from 2.72/100,000 in 2007 to 3.60/100,000 in 2017 (4). One nationwide registry study based on multiple centers showed that the incidence of T1DM in children aged between 0 and 14 years was 1.9/100,000 (5). Regional surveys in Beijing, Shanghai, and Zhejiang have found that the compound annual growth rate (CAGR) in T1DM incidence in China is much higher than the world average (6–8). Additionally, the CAGR in T1DM incidence in the children under 5 years old is approximately 5–34%, suggesting that the onset of the disease has an increasing trend at younger ages (9). The earlier age at onset of T1DM in childhood, the greater risk of dying from related acute and chronic complications. The average life expectancy of children with T1DM is reduced by about 12 years even in western developed countries (10, 11). However, there is a terrific lack of comprehensive information related to the clinical incidence and characteristics of T1DM in Chinese children and adolescents, particularly about the declining pancreatic β-cell function, T1DM-related antibodies, status of diabetic ketoacidosis (DKA), diagnosis, and treatment of the disease, etc. The aim of this study was to investigate these clinical features and test the hypothesis that children and adolescents who present with DKA at the diagnosis of T1DM have a higher antibody positive rate, worse pancreatic β-cell function, and need more efficient insulin therapy.

## Methods

### Participants

In a national observational cohort study from the National Diabetes Register, we recruited hospitalized patients <16 years of age with newly diagnosed T1DM from 34 medical centers in 25 major cities of China between January 2015 and January 2020. These cities include regions of different economic development levels throughout the country according to geographical location, climate, culture, and population (northeast, north, northwest, southwest, central, east, and south), involving 16 provinces, 2 autonomous regions, and 4 municipalities and covering 79.4% of the whole population of China. For the purpose of achieving a real representative profile of T1DM, all of the participating medical centers are the most influential ones in the local medical institutions attended by excellent pediatric endocrinologists. The inclusion criteria for T1DM were according to the standards of medical care in diabetes from American Diabetes Association (ADA) (12–14). DKA was defined by the International Society of Pediatric and Adolescent Diabetes (ISPAD) as follows: blood glucose above 200 mg/dl, metabolic acidosis (venous pH under 7.30 or serum bicarbonate under 15 mEq/L), and ketosis (ketonemia or ketonuria). According to venous pH, DKA can be classified into mild (under 7.30), moderate (under 7.20), or severe (under 7.10) (15, 16). Diabetic ketosis (DK) was defined as ketonemia and***/***or ketonuria. The present study excluded patients with an uncertain diagnosis of T1DM and those with diabetes secondary to some other conditions, such as hemochromatosis, cystic fibrosis, or chemotherapy. Participants were classified into three age groups: <5 years, 5 to <10 years, and ≥10 years. The same patient population was also categorized as DKA and non-DKA groups based on clinical criteria. Additionally, the patients with DKA were further grouped by the severity levels (mild/moderate/severe) for the study.

This study was approved by the University of Zhejiang's Institutional Review Board and adhered to the tenets of the Declaration of Helsinki. Written informed consent and assent, when appropriate, were obtained from all the participants or parents or legal guardians at the beginning of the study.

### Anthropometric and Laboratory Measures

The body weight and height of the participants were measured following a standardized procedure by trained investigators. The body mass index (BMI) was calculated as weight (kg)/height (m^2^) and was converted to BMI z score as [BMI–mean (BMI)]/SD (BMI), the mean (BMI) and SD (BMI) were obtained from the height and weight standardized growth charts of Chinese children and adolescents (17).

All subjects had blood drawn for plasma glucose, C-peptide (CP), glycated hemoglobin (HbA1c), pH, ketone bodies, serum bicarbonate along with T1DM-related antibodies, such as glutamic acid decarboxylase antibody (GADA), insulinoma antigen-2 antibody (IA−2A), insulin autoantibodies (IAA), Zinc Transporter 8 antibody (ZnT8A), and islet cell antibody (ICA). The urine sample was also collected for a test of glycosuria and ketonuria. Meanwhile, a questionnaire survey about the birth history and family history of diabetes was obtained. Insulin therapeutic regimens (continuous subcutaneous insulin infusion [CSII] with an insulin pump or multiple daily subcutaneous injections of insulin) were recorded and also both hospitalization and post-hospitalization durations.

### Statistical Analysis

Means (± SD), medians (interquartile range), or geometric means (95% CI) for continuous variables were calculated based on their distributions and proportions and were estimated for categorical variables. Continuous variables were compared using the independent sample t-test, Mann-Whitney U test, Univariate ANOVA, or Kruskal-Wallis test, depending on the distribution of variables and the numbers of groups compared. Categorical variables were compared using the chi-squared (**χ^2^**) test. The correlation analysis and partial correlation analysis were adopted for analyzing the relationship between variables. In the examination of <5 years, 5 to <10 years, and ≥10 years groups, we used analysis of covariance (ANCOVA) to compare the geometric means of adjusting for BMI z score as covariate, the ordinal logistic regression was used when the data distribution was skewed. In the study of DKA and non-DKA groups, the binary logistic regression was used, and age and BMI z score were used as independent variables. All comparisons were exploratory. A value of *p* < 0.05 was considered statistically significant. Statistical analysis was performed using IBM SPSS 20.0 statistical software.

### Clinical Incidence of T1DM

All newly diagnosed diabetes cases were outpatient or emergency department visits before hospitalization. Because the population of children and adolescents in all of the areas where the study centers located is not available, we cannot calculate the incidence of T1DM in the general population. However, the clinical incidence rate of T1DM, which reflects the incidence of disease in all children and adolescents attending clinical clinics, can be obtained. The clinical incidence rate of T1DM was calculated as the sum of newly diagnosed cases of both children and adolescents each year divided by the annual number of outpatient and emergency visits in 34 medical centers in China.

## Results

### Clinical Incidence of T1DM

**Figure 1:**
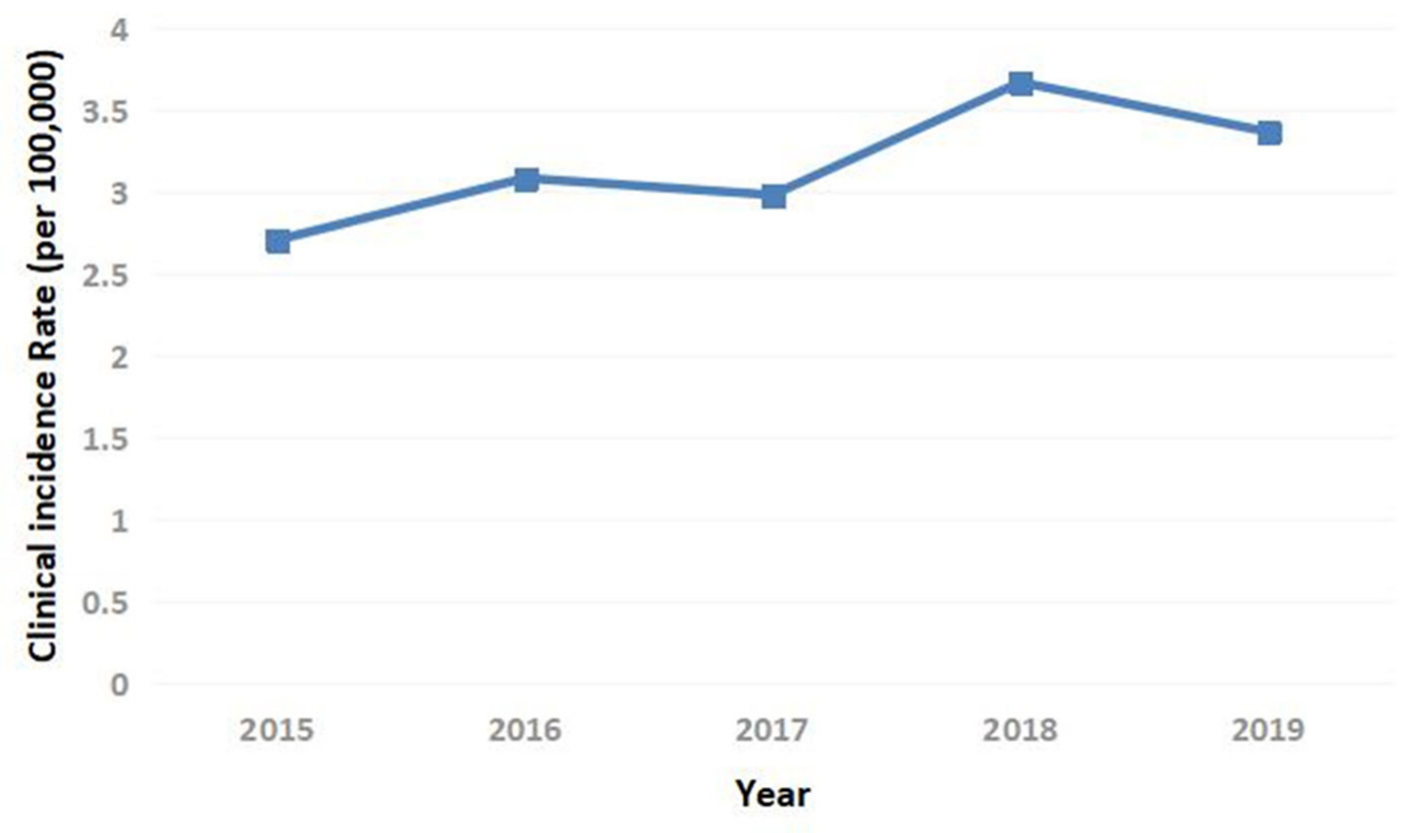
Clinical incidence of T1DM in children and adolescents in China.

**Figure 2:**
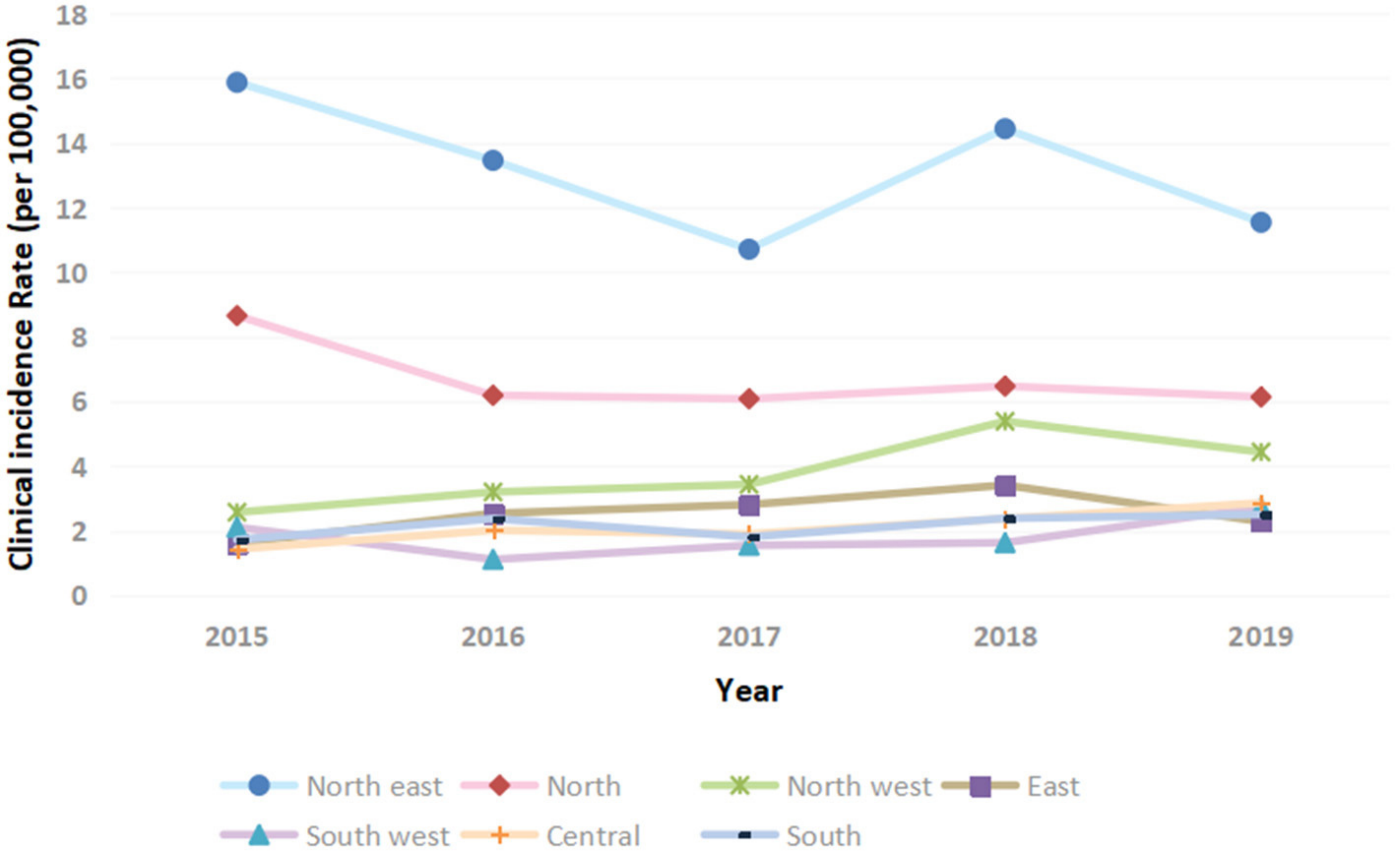
Clinical incidence of T1DM in children and adolescents in China stratified by geographical region.

The annual clinical incidence of T1DM had gradually increased from 2.71/100,000 in 2015, 3.08/100,000 in 2016, 2.98/100,000 in 2017, 3.67/100,000 in 2018 to 3.37/100,000 in 2019. Besides, within the country variation was seen with rates 6–8 times higher in the northeast than the southwest (Figures 1, 2). The mean annual clinical incidence of T1DM was 3.16/100,000, and the CAGR was 5.6% during the period 2015–2019.

### Participants Characteristics

In total, 7,700 patients aged 0–16 years were approached based on the inclusion criteria of T1DM. Of these, 7,206 patients were recruited. Among the participants, 34 were excluded because of the presence of diabetes secondary to some other conditions, such as hemochromatosis, cystic fibrosis, or chemotherapy. Additional 628 patients were excluded because of missing CP and/or T1DM-related antibody data. In the end, 6,544 patients with newly diagnosed T1DM were eligible for the study, with a median age of 7.84 ± 3.8 years, the proportion of each age group: <5 years (29.3%), 5 to <10 years (38.7%), and ≥10 years (32%), and 52.4% of women. The clinical characteristics of the participants were summarized as follows: BMI z score, −0.22 ± 1.58 (kg/m^2^); HbA1c, 11.8 ± 2.72 (%); pH of DKA, 7.15 ± 0.16; and CP, 0.25 ± 0.4 (ng/ml). Among the participants, the incidence rates of DK and DKA were 75.6 and 52.7%, respectively, at the first diagnosis. Overall 41.8% of the patients had measurable T1DM-related antibodies, such as GADA (27.7%), IA−2A (15%), IAA (13.2%), ZnT8A (10.4%), and ICA (9.6%); 19.8% of the patients showed two or more serum antibodies positive. In total, 90.5% of T1DM cases occurred in individuals without a family history of diabetes mellitus, only 2.1, 6.7, and 0.7% of the cases had a family history in first-, second-, or third-degree relatives, respectively. All of the newly diagnosed patients with T1DM received the treatment of insulin during their period of hospitalization. In total, 27.4% of them were treated with insulin pump (CSII) therapy, and the others (72.6%) were treated with multiple daily subcutaneous injections of insulin. However, a very low percentage (5.5%) of patients adopted insulin pump therapy post-hospitalization.

### Relationships Between the Clinical Parameters of Participants

There were noticeable positive correlations between CP and age, CP and BMI z score. CP had a weak correlation with the number of antibodies and family history, respectively. HbA1c was positively correlated with age, but it was inversely correlated with BMI z score. DKA status (yes/no) was positively correlated with the number of existing antibodies (r = 0.17, *p* = 0.000), while it was negatively correlated with CP. There was no noticeable correlation between HbA1c and CP (Table 1).

**Table 1:** Relationships between the clinical parameters of participants.

	**CP, ng/mL**	**HbA1c, %**
Gender, male/female	-	*-*
Age, years	r= 0.23, P <0.001^**a**^	r= 0.18, P <0.001^**a**^
BMI z score	r= 0.21, P <0.001^**b**^	r= −0.16, P <0.001^**b**^
DKA, yes/no	r= −0.18, P <0.001	-
Number of antibodies	r= −0.1, P=0.003	-
Family history, yes/no	r= 0.1, P <0.001	-

*Partial correlation analysis was used in analyzing the relationship of Age with CP and HbA1c, BMI z score with CP and HbA1c, the control variables were BMI z score (a) and Age (b) respectively. correlation coefficient: r; no correlation*.

### Comparative Analysis Among the age Groups

**Figure 3:**
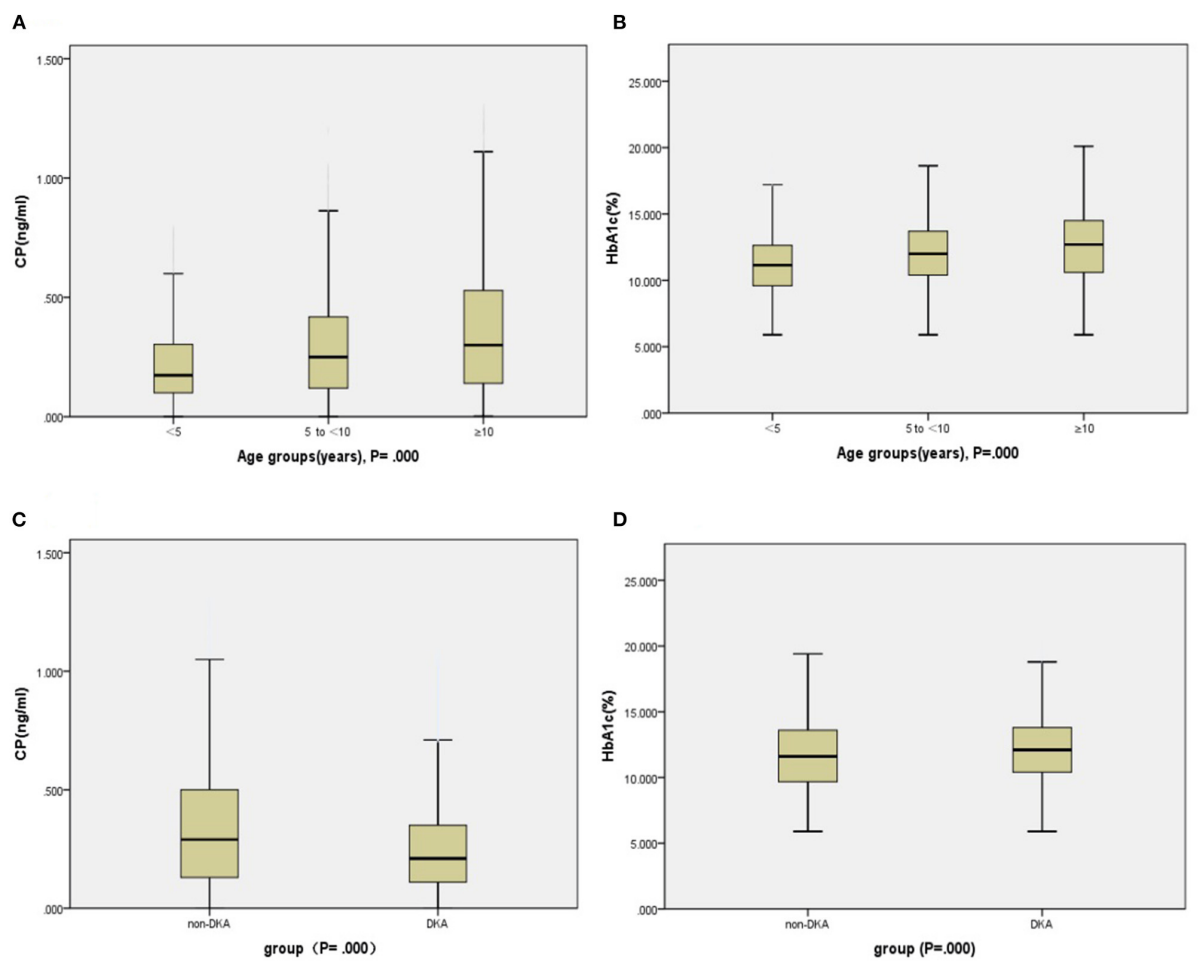
Indices of insulin secretion (CP) and HbA1c in the participants. Panels **(A,B)**: Age groups. Panels **(C,D)**: DKA and non-DKA groups.

Compared with the 5 to <10 years and ≥10 years groups, the <5 years group had significantly lower BMI z score, adjusted CP, and adjusted HbA1c (Figures 3A,B), and meanwhile, markedly higher the incidence of DK and DKA. Patients in the group of <5 years had a notably lower rate of CSII therapy during the period of hospitalization, while remarkably higher during the post-hospitalization sequential treatment when compared with the 5 to <10 years and ≥10 years groups. Although there was no significant difference in total antibody positive rates among the three age groups, the group of <5 years still had higher positive rates of GADA, IA−2A, and IAA than the others. There was no notably difference in family history among the three age groups (Table 2).

**Table 2:** Participant clinical parameters comparing <5 years, 5 to <10 years and ≥10 years.

	** <5 years**	**5 to <10 years**	**≥10 years**	***P*** **value**
Gender, female, %	49.7^**a**^	55	52	<0.01
BMI z score	−0.43 ± 0.15^**a, b**^	−0.17 ± 0.16	−0.16 ± 0.15	<0.001
HbA1c, %	11.27 ± 2.37^**a, b**^	11.97 ± 2.7^**c**^	12.47 ± 2.87	<0.001
CP, ng/mL	0.17 ± 0.03^**a, b**^	0.24 ± 0.04^**c**^	0.3 ± 0.08	<0.001
DK, yes, %	79.9^**a, b**^	72.3	75.2	<0.001
DKA, yes, %	56.9^**a, b**^	49.5	51.9	<0.001
GADA(+), %	30.2^**a**^	26.3	27.1	<0.05
IA-2A(+), %	17.8^**b**^	14.5	13.5	<0.05
IAA(+), %	16.4^**a, b**^	11.1	12.6	<0.001
ZnT8A(+), %	12.4	10.4	9	NS
ICA(+), %	11.6	9	9.9	NS
Total antibodies(+), %	42.8	43.3	38.8	NS
Family history(+), %	9.5	8.9	10	NS
Hospitalization CSII, %	22.4^**a, b**^	28.3	31.3	<0.001
Post-Hospitalization CSII, %	7.1^**a, b**^	5	4.6	<0.05

*NS, not significant*.

a * <5 years / 5 to <10 years*.

b * <5 years / ≥10 years*.

c *5 to <10 years / ≥10 years*.

*Data are mean (± SD). CP is median(M). The categorical variables (Gender, DK, DKA, GADA, IA-2A, IAA, ZnT8A*.

*ICA, Total antibody positive, Family history, Hospitalization and Post-Hospitalization CSII therapy) were presented as percentages(%). Values are derived from χ^2^ analysis for categorical variables and the Univariate ANOVA for quantitative variables. CP and HbA1c were derived from the ordinal logistic regression and one-way ANCOVA respectively, and P value was adjusted by BMI z score*.

### Comparative Analysis Between the DKA and Non-DKA Groups

**Figure 4:**
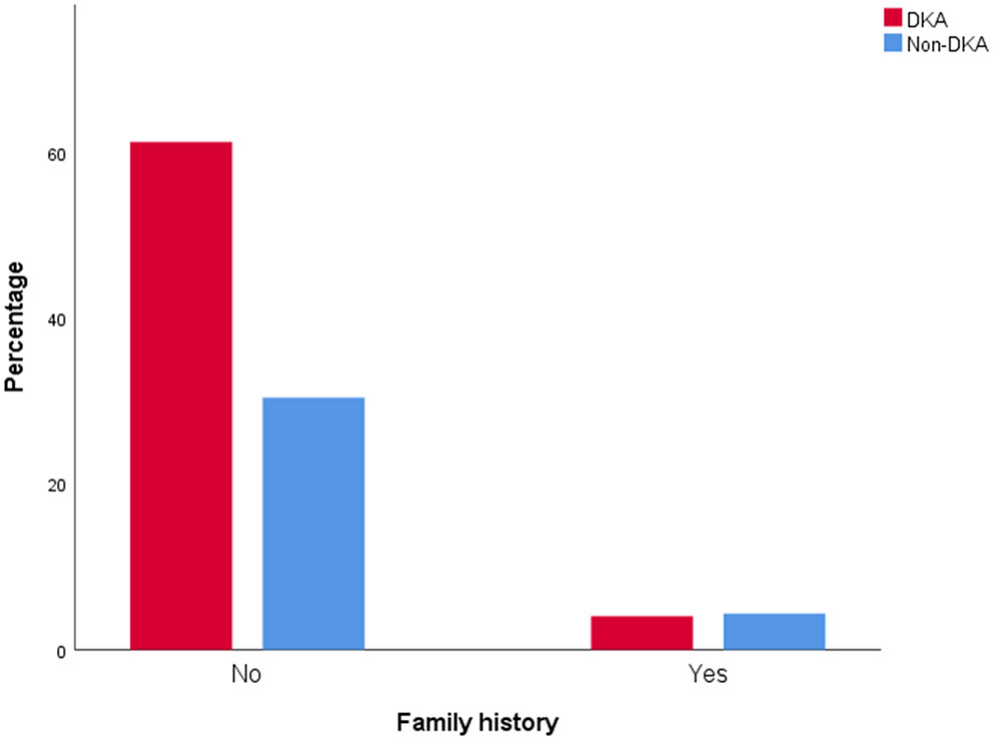
Percentage of family history positive in DKA and non-DKA groups respectively.

Compared with the non-DKA group, the DKA group had significantly lower age, BMI z score, CP (Figure 3C), and rate of family history positive (Figure 4), but in contrast, dramatically higher GADA, ICA, total antibodies positive rate, HbA1c (Figure 3D), and rate of CSII therapy. There was no notable difference in gender between the groups (Table 3).

**Table 3:** Participant characteristics and laboratory data comparing DKA and non-DKA.

	**DKA (*n* = 3449)**	**non-DKA (*n* = 3095)**	***P*** **-value**
Gender, female, %	51.53	52.9	NS
Age, years	7.83 ± 3.93	8.08 ± 3.66	0.01
BMI z score	−0.33 ± 0.15	−0.04 ± 0.14	<0.001
HbA1c, %	12.08 ± 2.61	11.61 ± 2.78	<0.001
CP, ng/mL	0.22 ± 0.33	0.32 ± 0.5	<0.001
GADA(+), %	29.8	26.4	<0.01
IA-2A(+), %	15.8	16.2	NS
IAA(+), %	13.4	13	NS
ZnT8A(+), %	10.1	12.3	NS
ICA(+), %	11	8.3	<0.01
Total antibodies(+), %	61.54	26.64	<0.001
Family history(+), %	6.2	12.6	<0.001
Hospitalization CSII, %	36.1	17.6	<0.001
Post-Hospitalization CSII, %	7.3	3.7	<0.001

*NS, not significant*.

*Data are mean (± SD). Age and CP are medians (M). The categorical variables (Gender, GADA, IA-2A, IAA, ZnT8A, ICA, Total antibody positive, Family history, Hospitalization and Post-Hospitalization CSII therapy) were presented as percentages(%). Values are derived from χ^2^ analysis for categorical variables and the independent sample T-test for quantitative variables. The Mann-Whitney U test was used when the data distribution was skewed. CP and HbA1c were derived from the binary logistic regression, P value was adjusted by age and BMI z score*.

Besides, there was no significant difference in gender, age, BMI z score, CP, HbA1c, total antibodies positive rate, family history, and the rate of CSII therapy among the DKA subgroups (mild, moderate, and severe groups).

## Discussion

Type 1 diabetes mellitus is a chronic autoimmune disease characterized by the loss of insulin-producing pancreatic β-cells. The pathogenesis of the disease is multifactorial and involves a genetic susceptibility that predisposes to abnormal immune responses in the presence of environmental triggers that insults to the pancreatic islets (18, 19). There is no single clinical feature, which can perfectly distinguish type 1 from non-type 1 diabetes at diagnosis. The classification depends on consideration of risk factors for type 1 vs. other subtypes and the integration of clinical features (e.g., age of diagnosis, BMI, and CP) with biomarkers (e.g., pancreatic autoantibodies) (20). In this cohort study, we found that the clinical incidence of T1DM in children and adolescents in China was 3.16/100,000, and it has been gradually increasing annually. That can indirectly reflect the incidence of T1DM in the general population. The peak period of disease onset was in school-aged children (aged 5 to <10 years, 38.7%), which is similar to the report of the SEARCH (SEARCH for Diabetes in Youth) study in the United States (21). Women had a slightly higher morbidity of T1DM, consistent with the previous findings (4), which may be one of the features of childhood T1DM in China. Although obesity is associated with the increasing presentation of T1DM for a potentially excess β-cell stress (22, 23), the patients were rarely obese at diagnosis in our study. We found that the patients were generally underweight with a lower BMI z score, furthermore, the BMI z score was negatively correlated with the severity of the disease. Despite T1DM is a heritable polygenic disease with identical twin concordance of 30–70%, sibling risk of 6–7%, and a risk of 1–9% for children who have a parent with diabetes (24, 25), the overwhelming majority of the cases occurred in individuals without a family history of diabetes mellitus (90.5%) in our study. It indicated that environmental risk factors may play a crucial role in the pathogenesis of T1DM in addition to genetic factors.

The pathogenesis of T1DM results from a complex interaction between the pancreatic β-cell and innate and adaptive immune systems, inflammation, and selective autoimmune destruction of the pancreatic β-cells resulting in insulin deficiency (26–28). The presence of autoantibodies is associated with the destruction of pancreatic β-cell. Previous studies have reported that over 90% of people with newly diagnosed T1DM have measurable antibodies, they are one of the most reliable biomarkers for autoimmune diabetes in both children and adults (29). In our study, overall 41.8% of the patients with newly diagnosed T1DM had measurable-related antibodies, and 19.8% of the total patients showed two or more serum antibodies positive. The rate of antibody positive is similar to Indian (30), but significantly lower than Caucasian, Japanese, and Korean (31, 32), the discrepancy may be due to the different levels of quality control in the test. Under the background of a low antibody positive rate, in order to avoid misdiagnosing other forms of diabetes among patients with negative autoantibodies, the classification should depend on consideration of risk factors for type 1 and the clinical features (e.g., age of diagnosis, BMI, and CP) and should get the genetic testing if necessary (e.g., it is difficult to distinguish from monogenic diabetes). We also found that patients aged <5 years had a higher positive rate of GADA, IA-2A, and IAA than the older age groups. Previous investigations have suggested that IAA is often the first expressed, especially in younger children, GADA positivity represents a propensity for general autoimmunity, while IA2-A positivity may be a more specific marker of β-cell destruction (19). It could be concluded that patients aged <5 years may be engaged in a very early autoimmunity leading to β-cell destruction and finally the onset of T1DM. People with T1DM have reduced β-cell function at diagnosis when compared with healthy ones (33, 34). Usually, low CP as a marker of severe endogenous insulin deficiency is useful to guide both classification and treatment in cases of diabetes (35). In this cohort, the β-cell function of participants has been severely impaired (reflected by a lower CP). Furthermore, we also find that younger age, lower BMI z score, and the presence of more antibodies are the destructive factors to the β-cell function. In addition, a worsening β-cell function means an increasing incidence of DKA.

Diabetic ketoacidosis is a serious complication of T1DM, which is characterized by the triad of hyperglycemia, acidosis, and ketosis caused by insulin deficiency. It is the main cause of morbidity and mortality in children and adolescents with T1DM due to the short-term risks and long-term consequences (36–39). Previous studies have shown that the frequency of DKA at diagnosis has ranged from 12.8% (Sweden) to 80% (United Arab Emirates), and it is inversely associated with gross domestic product, latitude, and background incidence of T1DM (40, 41). In our study, the incidence rate of DKA at the onset of T1DM was 52.7%, much higher than developed countries and similar to India (5, 42). It can be explained by different levels of disease awareness and healthcare provision, in countries with a low incidence of T1DM, the awareness of DKA tends to be low while the rate of DKA is high, conversely, in countries with higher incidence and prevalence rates of T1DM, people are more effective in the detection of symptoms of new-onset T1DM prior to DKA, and then many children with new-onset T1DM receive insulin therapy in time to avoid DKA. That also can be indirectly explained by the newly diagnosed patients with a family history of T1DM who usually had a low incidence of DKA. That suggests the ways to reduce the rate of DKA even further following appropriate campaigns, such as increased medical awareness and healthcare provision (43). We also find that factors associated with increased risk of DKA are younger age, lower BMI z score, lower CP, higher positive rate of antibodies, and the number of existing antibodies, while the protective factor is having a family history of T1DM at the time of diagnosis. Our finding is supported by a number of preceding observational studies (44, 45). Despite there was no significant correlation between HbA1c and DKA, the DKA group had a significantly higher HbA1c (11.8 ± 2.72, %) when compared with the non-DKA group. We have found that HbA1c is lower in younger patients in the study of age groups, but it is conversely higher in the younger patients with DKA, implying that young children with DKA at diagnosis already have more aggressive dysglycemia and β-cell destruction, which is associated with a low rate of partial remiss*ion* (37). Unfortunately, we found that the youngest patients (aged <5 years) had the highest incidence rate of DKA at the onset of T1DM (56.9%).

The Diabetes Control and Complications Trial (DCCT) showed that intensive glycemic control improved the outcomes in patients with T1DM (46, 47), and insulin therapy is the cornerstone. In this study, all of the newly diagnosed patients with T1DM received the treatment of insulin during the period of hospitalization, only 27.4% of them adopted insulin pump therapy (CSII), and the rate had plummeted to 5.5% during the post-hospitalization sequential treatment. The percentage of CSII therapy is much lower than in developed countries, such as the United States (50%), Germany, and Australia (74%) (48), that may be caused by multifactors that include economic factors, availability of resources, and patient's propensity to the treatment, etc. It has been previously reported that insulin pump therapy is more efficient in glycemic control and minimizes short- and long-term complications (49, 50). We also found that patients with DKA had a higher rate of CSII treatment both during hospitalization and post-hospitalization when compared with patients with non-DKA, indicating that patients with DKA need more efficient insulin therapy. DKA at diagnosis of T1DM in children predicts poor long-term glycemic control, increasing the risk for long-term complications. Targeted DKA prevention programs could result in substantial healthcare cost reduction and reduced patient morbidity and mortality (51). Public health measures for the prevention and treatment of T1DM should be directed toward the preschoolers (aged <5 years), considering the severity and highest frequency of DKA in this age group. Levels of disease awareness and healthcare provision should be improved timely. Previous studies have shown that immune-mediated β-cell destruction, marked by the presence of diabetes autoantibodies, occurs before and continues after the clinical diagnosis of T1DM, most patients will have complete destruction of β-cells within a few years after diagnosis without a targeted intervention to sustain β-cell function (28, 52). Multiple studies have reported that even the modest preservation of β-cell function could achieve better glycemic control and long-term benefits (53–55). Hence, more efforts should be dedicated to achieve early *screening and* diagnosis of the disease and preserve β-cell function as early as possible.

The primary limitation of this study is that as a multicenter study, the laboratory tests, such as HbA1c, autoantibody, and CP, were not measured in a central laboratory and this could introduce bias, although all the tests were performed using a standardized assay protocol. Furthermore, differences in race/ethnicity factors among the participants may explain some of the outcome differences, though the number of participants of racial and ethnic minorities is very small, we cannot make a comparative study between different ethnic groups due to the lack of relevant data. Further research studies are urgently required.

## The T1DM China Study Group for Children and Adolescents

Guo-Hua Li, Ke Huang, Guan-Ping Dong, Jian-Wei Zhang, Chun-Xiu Gong, Fei-Hong Luo, Xiao-Ping Luo, Chun-Lin Wang, Min Zhu, Pin Li, Zhi-Ya Dong, Hua-Mei Ma, Li Liu, Hong-Wei Du, Lan-Wei Cui, Yu Yang, Zhi-Hua Wang, Gui-Mei Li, Xin-Ran Cheng, Mireguli.Maimaiti, Zhe Su, Hai-Yan Wei, Rong-Xiu Zheng, Ying Xin, Tang Li, Rui-Min Chen, Lin-Qi Chen, Fan Yang, Shao-Ke Chen, Yu-Qing Chen, Ying-Xue, Yu-Liu, Ling Wang, Jun-Fen Fu^*^

Guo-Hua Li, Ke Huang, Guan-Ping Dong, Jun-Fen Fu^*^, Department of Endocrinology, The Children's Hospital of the Zhejiang University School of Medicine, National clinical research center for child health, Hangzhou, China. Jian-Wei Zhang, Department of Pediatrics, Shaoxing Women and Children's Hospital, Hangzhou, China. Chun-Xiu Gong, Department of Endocrinology, Genetics, and Metabolism, Beijing Diabetes Center for Children and Adolescents, Medical Genetics Department, Beijing Children's Hospital, Beijing, China. Fei-Hong Luo, Department of Pediatric Endocrinology and Inherited Metabolic Diseases, Children's Hospital of Fudan University, Shanghai, China. Xiao-Ping Luo, Department of Pediatrics, Tongji Hospital, Tongji Medical College, Huazhong University of Science and Technology, Wuhan, China. Chun-Lin Wang, Department of Pediatric, The First Affiliated Hospital of Zhejiang University School of Medicine, Hangzhou, China. Min Zhu, Department of Endocrinology, Children's Hospital of Chongqing Medical University, Chongqing, China. Pin Li, Department of Endocrinology, Shanghai Children's Hospital of Shanghai Jiao Tong University, Shanghai, China. Zhi-Ya Dong, Department of Pediatrics, Ruijin Hospital, Shanghai Jiao Tong University, Shanghai, China. Hua-Mei Ma, Department of Pediatrics, The First Affiliated Hospital of Sun Yat-sen University, Guangzhou, China. Li Liu, Department of Genetics and Endocrinology, Guangzhou Women and Children's Medical Center, Guangzhou, China. Hong-Wei Du, Department of Pediatric Endocrinology, The First Bethune Hospital of Jilin University, Changchun, China. Lan-Wei Cui, Department of Pediatric, The First Affiliated Hospital of Harbin Medical University, Harbin, China. Yu Yang, Department of Endocrinology, Children's Hospital of Nanchang University & Jiangxi Provincial Children's Hospital, Nanchang, China. Zhi-Hua Wang, Department of Endocrinology, Xi'an Children's Hospital, Xi'an, China. Gui-Mei Li, Department of Pediatric, Shandong Provincial Hospital, Jinan, China. Xin-Ran Cheng, Department of Endocrinology, Chengdu Women's and Children's Central Hospital, Chengdu, China. Mireguli. Maimaiti Department of Pediatric, The First Affiliated Hospital of Xinjiang Medical University, Urumqi, China. Zhe Su, Department of Endocrinology, Shenzhen Children's Hospital, Shenzhen, China. Hai-Yan Wei, Department of Pediatric Endocrinology and Inherited Metabolic Diseases, Henan Children's Hospital, Zhengzhou, China. Rong-Xiu Zheng, Department of Pediatric, Tianjin Medical University General Hospital, Tianjin, China. Ying Xin, Department of Endocrinology, Shengjing hospital of China Medical University, Shenyang, China. Tang Li, Department of Pediatrics, Qingdao women and children's hospital, Qingdao University, Qingdao, China. Rui-Min Chen, Department of Endocrinology, Children's Hospital of Fuzhou, Fujian Province, Fuzhou, China. Lin-Qi Chen, Department of Pediatric Endocrinology, Children's Hospital Affiliated to Soochow University, Suzhou, China. Fan Yang, Department of Pediatrics, West China 2nd University Hospital, Sichuan University, China. Shao-Ke Chen, Department of Pediatrics, The Second Affiliated Hospital of Guangxi Medical University, Nanning, China. Yu-Qing Chen, Department of Endocrinology and Rheumatology, Anhui Provincial Children's Hospital, Hefei, China. Ying Xue, Department of Pediatric Endocrinology, Xuzhou Children's Hospital, Xuzhou, China. Yu Liu, Department of Pediatric Endocrinology and Inherited Metabolic Diseases, Guiyang Maternal and Child Health Hospital, Guiyang, China. Ling Wang, Laboratory for Translational Genetics, Department of Human Genetics, KU Leuven, Leuven, Belgium. These authors contributed equally to the study. The T1DM China Study Group for Children and Adolescents is officially authorized by the Bureau of Medical Administration, Inspection and Supervision of National Health and Family Planning Commission of the People's Republic of China, funded by China International Medical Foundation, and supervised by the Chinese Medical Association.

## Data Availability Statement

The original contributions presented in the study are included in the article/supplementary material, further inquiries can be directed to the corresponding author.

## Ethics Statement

The studies involving human participants were reviewed and approved by University of Zhejiang's Institutional Review Board. Written informed consent to participate in this study was provided by the participants' legal guardian/next of kin.

## Author Contributions

G-HL and J-FF conceptualized and designed the clinical study. KH, G-PD, J-WZ strengthened the coordination and development of the project among various research centers. G-HL accomplished the data collection and analysis with the assistance of LW, and wrote the manuscript. All authors were responsible for the project carried out in their own centers. All authors contributed to the article and approved the submitted version.

## Funding

This research was supported by the project of Prevalence and Risk of Obesity and Diabetes in Youth (PRODY) and was funded by the National Key Research and Development Program of China (no. 2016YFC1305301), the National Natural Science Foundation of China (no. 81570759), the Fundamental Research Funds for the Central Universities (2020XZZX002-22), the Research Fund of Zhejiang Major Medical and Health Science and Technology & National Ministry of Health (WKJ-ZJ-1804), Zhejiang Provincial Key Science and Technology Project (LGF21H070004), Zhejiang Provincial Natural Science Foundation of China [grant number LQ20H070003], and Zhejiang Province Natural Sciences Foundation Zhejiang Society for Mathematical Medicine (LSZ19H070001). The effect of intestinal flora and its metabolites on the changes in islet function in children with type 1 diabetes mellitus during the honeymoon (no. LGF21H070004).

## Conflict of Interest

The authors declare that the research was conducted in the absence of any commercial or financial relationships that could be construed as a potential conflict of interest.

## Publisher's Note

All claims expressed in this article are solely those of the authors and do not necessarily represent those of their affiliated organizations, or those of the publisher, the editors and the reviewers. Any product that may be evaluated in this article, or claim that may be made by its manufacturer, is not guaranteed or endorsed by the publisher.

## Abbreviations

T1DM: type 1 diabetes mellitus
CP: C-peptide
CAGR: compound annual growth rate
DKA: diabetic ketoacidosis
DK: Diabetic ketosis
BMI: body mass index
HbA1c: glycated hemoglobin
GADA: glutamic acid decarboxylase antibody
IA-2A: insulinoma antigen-2 antibody
IAA: insulin autoantibodies
ZnT8A: Zinc Transporter 8 antibody
ICA: islet cell antibody
CSII: continuous subcutaneous insulin infusion.
